# Supplementary material for: Comparative genomics provides new insights into the diversity, physiology, and sexuality of the only industrially exploited tremellomycete: Phaffia rhodozyma
Source: BMC Genomics. 2016 Nov 9;17:901. doi: 10.1186/s12864-016-3244-7 (PMC5103461; doi:10.1186/s12864-016-3244-7)
Supplement: Additional file 6: — List of orphan genes with links to PFAM (related to Additional file 1: Table S1). (ZIP 1428 kb) [file 12864_2016_3244_MOESM6_ESM.zip › PFAM_UNIQUE/G03952_P.html]

#                                                                            --- full sequence --- -------------- this domain -------------   hmm coord   ali coord   env coord
  
# target name        accession   tlen query name           accession   qlen   E-value  score  bias   #  of  c-Evalue  i-Evalue  score  bias  from    to  from    to  from    to  acc description of target
  
#------------------- ---------- ----- -------------------- ---------- ----- --------- ------ ----- --- --- --------- --------- ------ ----- ----- ----- ----- ----- ----- ----- ---- ---------------------
  
G03952\_P             -           1204 CENP-B\_dimeris       PF09026.5    101       0.3   10.0  11.6   1   1    0.0049      0.86    8.6  11.6     7    37   125   155   120   166 0.76 - Centromere protein B dimerisation domain  
G03952\_P             -           1204 DUF4611              PF15387.1     96       1.2    8.0   7.4   1   1     0.024       6.2    5.7   7.4    58    89   129   160   109   167 0.73 - Domain of unknown function (DUF4611)  
G03952\_P             -           1204 NOA36                PF06524.7    314       3.9    5.3   8.0   1   1     0.032         7    4.5   8.0   251   298   109   155    97   167 0.61 - NOA36 protein  
G03952\_P             -           1204 TLP-20               PF06088.6    169     0.063   11.6   0.9   1   1   0.00038      0.28    9.5   0.9    89   147    97   154    86   170 0.55 - Nucleopolyhedrovirus telokin-like protein-20 (TLP20)
